# Experimental Immigration Mediates Ecological Selection and Drift in Monarch Microbiome Assembly

**DOI:** 10.1111/ele.70252

**Published:** 2025-11-09

**Authors:** Christopher P. Catano, James G. DuBose, Lydia Fuller‐Hall, Joselyne Chavez, Jacobus C. de Roode

**Affiliations:** ^1^ Department of Botany & Plant Sciences University of California Riverside Riverside California USA; ^2^ Department of Biology Emory University Atlanta Georgia USA; ^3^ Department of Ecology, Evolution, and Organismal Biology Brown University Providence Rhode Island USA

**Keywords:** alpha & beta diversity, determinism & stochasticity, dispersal, gut bacterial community, host‐microbe symbiosis, lepidoptera— *Danaus plexippus*, species pool

## Abstract

The distribution of biodiversity depends on processes operating across scales, yet multiscale paradigms have struggled to permeate host‐microbiome research. Instead, host‐microbiome research has focused on host selection and has struggled to explain the high variation in microbial composition across individuals. By integrating multi‐scale ecological theory with experimental manipulation of bacteria colonizing monarch butterfly caterpillars, we test the hypothesis that immigration from the regional species pool alters the importance of niche selection and drift in causing variation in gut bacterial communities across individuals and through ontogeny. Higher immigration increased the dominance of certain bacteria, causing greater convergence in bacterial composition across the caterpillar life stage. Conversely, limited immigration made colonization more stochastic, resulting in more unpredictable variability in bacterial composition across individuals. Our study reveals that immigration mediates the balance between host selection and drift, demonstrating that processes operating at scales beyond the individual are underappreciated but critical for structuring host‐microbiome symbioses.

## Graphical abstract

The distribution of biodiversity depends on processes operating across scales, yet multiscale paradigms have struggled to permeate host‐microbiome research. By integrating multi‐scale ecological theory with experimental manipulation of bacteria colonizing monarch butterfly caterpillars, we test the hypothesis that immigration from the regional species pool alters the importance of niche selection and drift in causing variation in gut bacterial communities across individuals and through ontogeny. Our study reveals that immigration mediates the balance between host selection and drift, demonstrating that processes operating at scales beyond the individual are underappreciated but critical for structuring host‐microbiome symbioses.

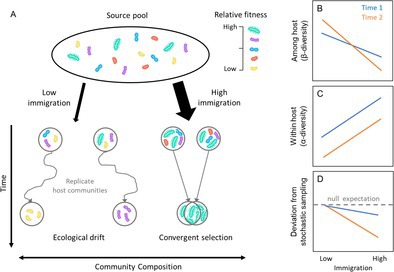

## 1 Introduction

Understanding the processes that structure the distribution of biodiversity is the fundamental challenge of ecology, but despite a rich history, ecologists have struggled to reveal general laws (Lawton 1999). The Theory of Ecological Communities provides a conceptual synthesis where all biodiversity change emerges from four general processes—diversification, dispersal, ecological drift and niche selection (Vellend 2016; Vellend 2010). The importance of these processes has been revealed through studies of macroorganisms (Catano et al. 2017; Ron et al. 2018; Gilbert and Levine 2017) and free‐living microbes (Stegen et al. 2013; Ferrenberg et al. 2013); however, their role in structuring the assembly of host‐associated microbial communities remains limited (Dini‐Andreote and Raaijmakers 2018; Miller et al. 2018; Kohl 2020). Moreover, it remains unclear whether host‐associated microbial communities are constrained by similar processes as macroorganisms because rapid population growth, horizontal gene transfer and feedbacks with their hosts (Ezenwa et al. 2012) can contribute to surprisingly high variation in microbial composition among individuals (Falony et al. 2016). Resolving the causes of this variation is a priority for understanding the extent to which ecological theory generalises across domains of life (Härer and Rennison 2023; Nemergut et al. 2013), the consequences of host microbiomes for broad‐scale ecological and evolutionary divergence (Moran et al. 2019; McFall‐Ngai et al. 2013) and to inform applications for human health (Gilbert et al. 2018; Mazmanian et al. 2008; Bäckhed et al. 2005).

Two interrelated issues that limit understanding of why host microbial communities are highly variable are an emphasis on host selection and mechanisms that operate at the scale of the individual. First, host selection is generally invoked to explain why individuals share similar microbiota, for example due to coevolution between hosts and their bacterial symbionts (Brooks et al. 2016), or filtering by the gut environment (Mazel et al. 2018). Likewise, divergence in microbiota across individuals is often attributed to factors such as differences in host genetics, diet (Nielsen et al. 2023) or immunity (Vega and Gore 2017; Taylor and Vega 2021). While studies are beginning to recognize the role of drift (Venkataraman et al. 2015; Burns et al. 2016), it remains unclear why the relative importance of niche selection and drift varies across individuals or through ontogeny. Second, studies historically have not considered the importance of processes that operate at scales larger than individual hosts, especially immigration from external species pools (Miller et al. 2018). One study that limited natural dispersal in nectar yeast communities found that higher dispersal increased variation in microbial composition across host plants (Vannette and Fukami 2017), in contrast to evidence from plant communities (Catano et al. 2017; Catano et al. 2025) and predictions from community ecology theory (Leibold et al. 2004; Mouquet and Loreau 2003). Therefore, to advance our understanding of the causes of variation in host‐microbiome assembly we need an emphasis on experiments that manipulate the multiscale processes thought to structure variation in microbial communities within and across hosts (Cecala et al. 2025).

**FIGURE 1:**
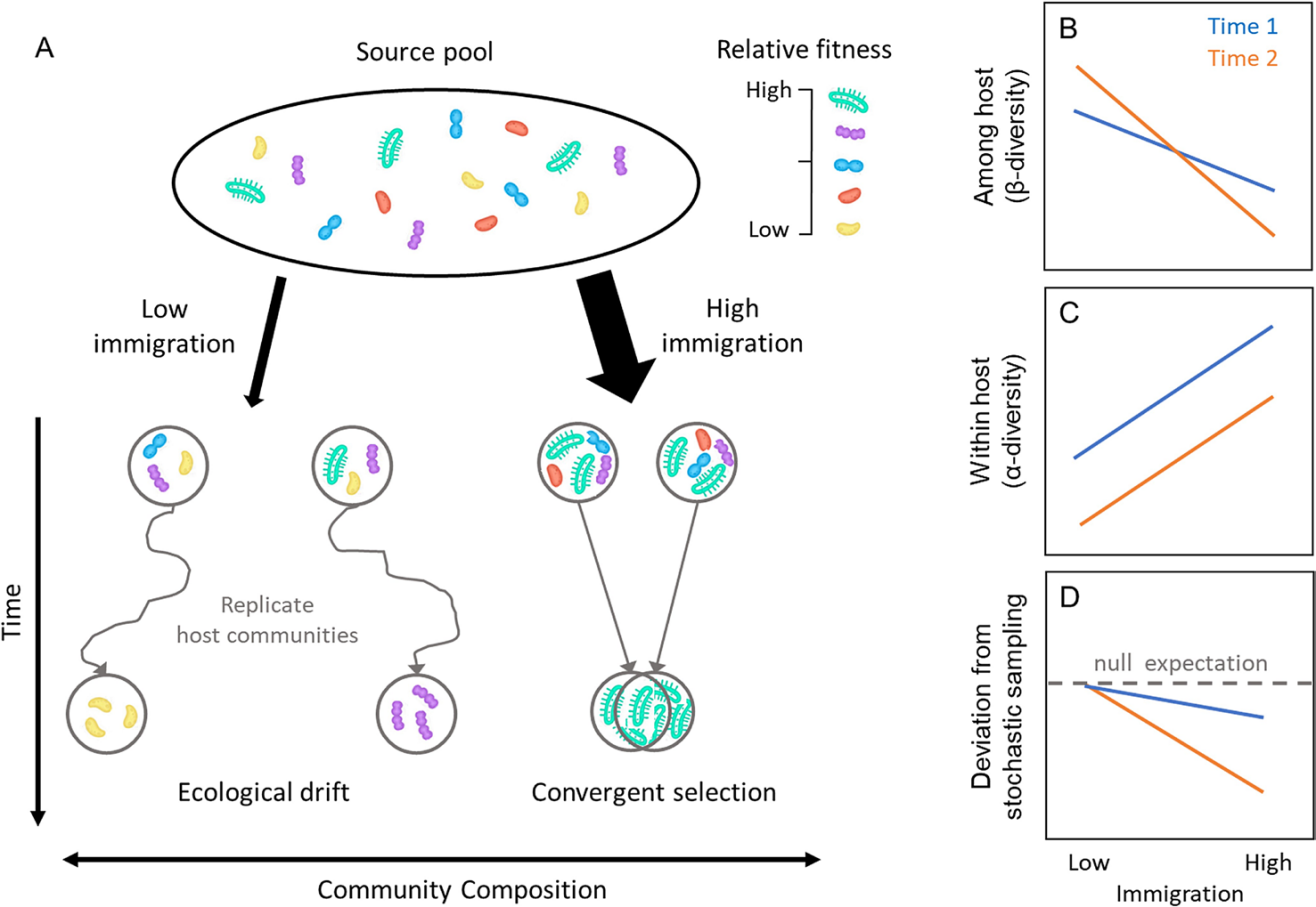
Conceptual diagram of how immigration alters community composition (A) and patterns of bacterial diversity among (B) and within (C) individual hosts. Low immigration can cause drift when the superior species in the pool (e.g., that with the highest fitness) does not establish and/or all species establish with low initial population sizes that make outcomes more susceptible to chance extinctions. High immigration can increase the role of deterministic outcomes based on differences between individuals of different species, leading to dominance by the species with the highest fitness. Selection will cause patterns of compositional variation among individuals that are greater or less than expected from stochastic sampling (D).

Here, we test the hypothesis that bacterial immigration—the arrival of new individuals from an external species pool (Mouquet and Loreau 2003)–mediates the relative importance of niche selection and drift (Vellend et al. 2014) in structuring microbial assembly within and among individual hosts. Niche selection is the change in bacterial relative abundances that are deterministic with respect to bacterial identity (Vellend 2016), whereas drift results from fluctuations in relative abundance that are stochastic with respect to bacterial identity (Hubbell 2001). Low immigration can increase the relative importance of drift through two, non‐mutually exclusive mechanisms. First, low immigration can make colonisation more probabilistic and reduce the likelihood that strong competitors establish in a host, thus mitigating competition (Hurtt and Pacala 1995; Orrock and Fletcher Jr. 2005). Second, low immigration can cause bottlenecks in initial community size (e.g., the bacterial load in the gut) of arriving taxa, or fail to rescue taxa with low population sizes (Mouquet and Loreau 2003), thus promoting drift due to demographic stochasticity (Vellend et al. 2014) (Figure 1A). The community size hypothesis, which is adapted from classic population genetics theory, predicts the importance of drift is inversely proportional to the number of individuals in a community (Hubbell 2001; Hu et al. 2006). Therefore, low immigration that increases drift is predicted to (1) constrain local community size, (2) increase compositional divergence across individual hosts (β‐diversity) (Figure 1B) and (3) erode within‐host (*α*) diversity due to probabilistic extinctions (Figure 1C).

In contrast, higher immigration is hypothesized to increase the relative importance of niche selection by increasing the number of individuals necessary for deterministic outcomes of competition (the effective community size) (Ron et al. 2018; Hu et al. 2006; Orrock and Watling 2010). For example, increasing immigration is predicted to increase the establishment of the superior competitor from the regional pool (Figure 1A), thereby leading to greater competitive exclusion that erodes bacterial diversity within hosts (α‐diversity) and homogenises composition among hosts (reduce β‐diversity) (Figure 1B,C) (Mouquet and Loreau 2003). In this scenario, homogenising niche selection generates patterns of microbial β‐diversity that are lower than expected from randomly sampling individuals from the regional pool (Chase and Myers 2011; Kraft et al. 2011) (Figure 1D). However, if immigration is very high it could swamp signals of selection (i.e., mass effect), thereby increasing α‐diversity within hosts due to higher colonisation from the pool (Leibold et al. 2004; Mouquet and Loreau 2003). These hypotheses make clear that the balance of niche selection and drift depends on immigration, yet because the prevailing view in microbial ecology is that ‘everything is everywhere–the environment selects’ (Baas‐Becking 1934), dispersal processes are rarely quantified (Nemergut et al. 2013). However, broad‐scale studies show ‘everything’ is not ‘everywhere’–microbes often have biogeographic affinities not explained by current environments, variation in dispersal abilities and differences in population density–which contributes to spatial structure that can alter colonisation and community assembly processes (Martiny 2006; Fierer and Lennon 2011). To date, the relatively few host‐microbiome studies that evaluate the role of dispersal typically do so using indirect proxies, like spatial distance (Belisle et al. 2012) or passive transmission between hosts housed together (Burns et al. 2017). Experiments that directly manipulate dispersal (Vannette and Fukami 2017; Cecala et al. 2025) are needed to quantify its role in mediating the importance of niche selection and drift in the assembly of host‐associated bacterial communities (Miller et al. 2018).

We designed an experiment to quantify the extent to which bacterial immigration mediates niche selection and drift structuring variation in host‐associated bacterial communities. Our experiment occurs in the context of gut microbial assembly in a non‐model holometabolous organism—the larvae of monarch butterflies, *Danaus plexippus* —thus providing an opportunity to test fundamental ecological theory with an unusual degree of realism for host‐associated microbiotas. We manipulated bacterial immigration over six orders of magnitude into individual caterpillars reared on their host milkweed, *Asclepias incarnata* , in a greenhouse, then quantified changes in bacterial composition and abundance through ontogeny (across developmental stages). We tested key predictions of the hypothesis that lower immigration reduces the importance of niche selection relative to drift by decreasing the effective community size (illustrated in Figure 1); where drift is predicted to (1) erode bacterial diversity within individual hosts; (2) increase divergence in bacterial composition across individuals through ontogeny; and (3) produce patterns of compositional variation that do not deviate from a null model of stochastic assembly.

## 2 Materials and Methods

### 2.1 Microbial Mixes and Experimental Community Assembly

To manipulate bacterial immigration magnitude, we created six immigration treatments composed of the same five bacterial taxa in equal relative proportions (therefore controlling compositional variation of the initial immigrant assemblage), where the total number of bacteria varied across treatments by six orders of magnitude. First, we isolated bacterial colonies, which represent clones of individual bacterial strains, from the frass of 4th instar monarch caterpillars reared on potted swamp milkweed ( *Asclepias incarnata* ) in a greenhouse under controlled conditions. Prior research in our lab demonstrates that bacteria isolated from frass are representative of the dominant taxa represented in the caterpillar gut (Sanaei et al. 2024); therefore, the bacteria we used to inoculate caterpillars in our experiment are those that occur in the caterpillar gut and have the potential to interact. Then we sequenced the 16S rRNA gene (27f/1492r) of the isolates, which allowed us to confidently identify them to genus (Frank et al. 2008; Monciardini et al. 2002). Based on prior correlative data from our lab, we selected isolates from five families that we have consistently observed to vary in relative abundance and across instars within the gut of monarch caterpillars (Sanaei et al. 2024): *Enterobacteriaceae* (*Enterobacter* sp.), *Comamonadaceae* (*Delftia* sp.), *Bacillaceae* (*Bacillus* sp.), *Xanthomonadacea*e (*Stenotrophomonas* sp.) and *Pseudomonadaceae* (*Pseudomonas* sp.). Differences in relative abundance represent potential differences in growth and survival across taxa, thereby allowing the possibility of niche‐selection to cause changes in bacterial composition across treatments. The specific source of these differences could be generated by specialised adaptation to the phyllosphere environments and the prevailing view is that the composition of caterpillar guts simply reflects immigration from their host plant (Hammer et al. 2017). The openness of caterpillars to bacterial immigration from host plants suggests that competition‐colonisation trade‐offs could be relatively unimportant and that all bacteria should be similarly likely to colonise the host.

For each family, we then inoculated aliquots of 1‐mL Sterile RPI Nutrient Broth with one colony of each bacterial isolate. The cultures were stored at 28°C shaking at 300 rpm and allowed to grow for 18 h. We diluted the liquid cultures to 10^6^ CFU/μL in 1 × PBS buffer (pH 7.5) and combined them to create the microbe mixture. The concentration of the final microbe mixture was 1.69 × 10^5^ CFU/μL and served as the concentration of the highest immigration treatment. We created the remaining immigration treatments by diluting this mix over five orders of magnitude (10^−1^ ➔ 10^−5^). Bacterial densities for lepidopterans were estimated from one study to range from 10^3^ to 10^7^ (16S rRNA copies per gram of faeces) (Hammer et al. 2017), therefore, our immigration treatments span the upper range of natural variation. Spanning these extremes from low to high densities provides an opportunity to assess potential outcomes typically observed and those that might occur under more extreme environmental conditions, such as drought or other disturbances. Finally, we randomly assigned caterpillars to treatments, and following methods similar to other studies inoculating caterpillars with microbial mixes (Phalnikar et al. 2019), we fed 3rd instar caterpillars leaf disks of *A. incarnata* with 2‐μL doses of their respective bacterial mixtures or control leaf disks that received 2‐μL of sterile PBS. Therefore, all caterpillars receive phyllosphere microbes while immigration treatments increase the arrival and initial abundance of the focal bacterial taxa. Feeding caterpillars leaf disks at the 3rd instar is necessary to ensure they are large enough to reliably consume the entire leaf disk while they are still early in development (day three of the caterpillar's life). To ensure treatments reached the specified bacterial densities and relative abundance we prepared mixes and dilutions fresh the day of inoculation, measured OD_600_ to confirm concentrations and plated 50‐μL of each mix for colony counts. We started with 200 caterpillars, but only those that consumed their leaf disk within 4 h were retained for processing and analysis (*n* = 92; 5–9 replicate caterpillars per immigration × instar treatment combination).

We inoculated caterpillars (obtained from four full‐sib lab‐reared lineages, as descendants from wild‐caught monarchs in October 2022) from all treatments at the beginning of the 3rd instar; then half were dissected and sequenced 24 h later to determine initial bacterial establishment and composition. Sampling 24 h after inoculation provides a balance between sampling long enough after ingestion to minimize the chance that microbes detected are not simply passing through the digestive tract on leaf material, but short enough to ensure we can sample individuals before they shed their gut lining and progress to the next development stage (instar). The remaining caterpillars were reared on potted *A. incarnata* plants held in clear plastic tubes (11 cm diameter × 60 cm height; net on top) in the greenhouse until the 5th instar (Day 13 and the last stage of caterpillar development), when their midguts were sequenced. Quantifying patterns of bacterial convergence and divergence across almost the entire caterpillar life stage enables us to test key predictions of drift and niche selection that emerge during and post colonization. This temporal range is extremely rare in manipulative experiments of host‐associated microbiomes, especially holometabolous organisms like lepidopterans.

### 2.2 Quantifying Bacterial Community Composition and Diversity

We determined caterpillar gut bacterial community composition and changes over time by sequencing bacterial DNA from caterpillar midguts. Library preparation was performed by the University of Georgia Genomics Core using the 341f and 785r primers, which target the V3–V4 region of the 16S rRNA gene (Klindworth et al. 2013). Raw sequences can be accessed from the NCBI SRA database and using the BioProject ID PRJNA1106479. However, sequencing error rates limited our capacities to accurately quantify the abundance of ASVs representing focal taxa when rare, which is especially likely in the lowest immigration treatments. Therefore, we use a more conservative approach by aggregating bacteria to the family level (ASVs representing isolates of focal bacteria represented 77% of the reads within their respective families across treatments). We performed all sequence processing using QIIME2 (v. 2023.2). See Supporting Information: Methods for the full DNA sequencing and processing methods.

**FIGURE 2:**
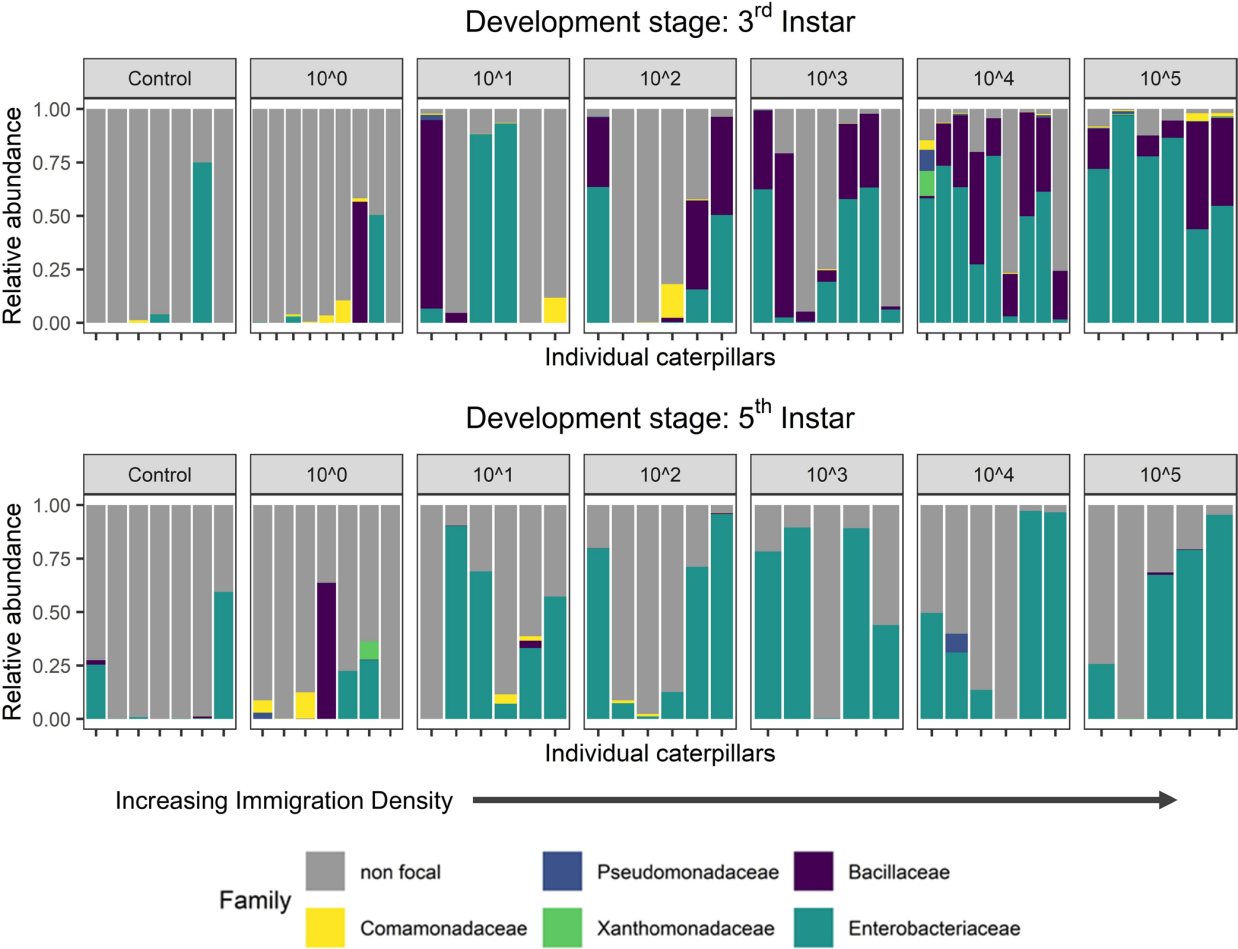
Variation in gut bacterial community composition among individual caterpillars by treatments: Immigration density (×1.7 CFU/μL) and Time (larval development stage; 3rd [*n* = 50] vs. 5th instar [*n* = 42]). Named families are those included in treatment inoculations and non‐focal taxa are those that colonised naturally. Control individuals were fed leaf disks from the host plant and inoculum solution without added bacteria.

We confirmed that the microbial mixtures we created introduced a novel assemblage of bacteria within individual caterpillars, with focal taxa persisting through development. Of the bacteria we added, ASVs of Enterobacteriaceae were detected at > 25% relative abundance in > 90% of caterpillars in the high immigration treatments compared to < 19% of control caterpillars; no other taxa that we added with our treatments occurred in control caterpillars at > 1% relative abundance (Figure 2). Also, the total proportion of bacteria in the gut comprised of the focal taxa we added increased as expected with immigration, reaching average proportions of 95% (0.05 std. dev) in 3rd instar caterpillars and 54% (0.29 std. dev.) in 5th instar caterpillars (Table S1). In contrast, focal bacteria averaged only 11% (0.28 std. dev.) and 13% (0.23 std. dev.) across control caterpillars at the 3rd and 5th instars, respectively.

We quantified three key response variables for the five focal bacteria added in the immigration treatments to test predictions of selection and drift: gut community size (bacterial abundance in each caterpillar: 16S rRNA bacterial copy number per μL of extracted DNA using real‐time qPCR; Supporting Information: Methods), β‐diversity (variation in gut bacterial composition among individual caterpillars) and α‐diversity (gut bacterial diversity within individual caterpillars). We quantified α‐diversity as both richness (number of bacterial families) and the effective number of families using the inverse of Simpson's Dominance. These diversity metrics are derived from the same general equation where the importance of relative abundance is weighted according to an exponent (*q* = 0 and 2, respectively). Using both diversity metrics allows us to assess treatment effects due to changes in bacterial incidence and abundance (Jost 2006), which is important to assess effects of drift and selection that are predicted to increase extinction/dominance (i.e., more uneven relative abundances). We measured variation in microbial composition among individual caterpillars within each Immigration treatment × Instar (development stage) combination using relative abundance‐weighted Bray–Curtis dissimilarity. Because raw dissimilarity values are not independent and therefore cannot be modelled directly, we first quantified β‐diversity as multivariate dispersion—the distance of each individual's gut community to the centroid (spatial median in principal coordinate space) of all individuals in each Immigration treatment × Instar combination (Anderson 2006). Small sample sizes and negative squared distances can force β‐diversity estimates to zero; therefore, we applied a $\sqrt{n/\left(n-1\right)}$ small sample bias correction and square root adjustment using the betadisper() function with arguments (bias.adjust = TRUE and sqrt.dist = TRUE) in vegan v2.6‐4 (Oksanen et al. 2022). Increasing dispersion from 3rd to 5th instar indicates bacterial compositions within a treatment are diverging, whereas decreasing dispersion indicates compositions are becoming more similar (Anderson et al. 2011). For metrics of α‐diversity and β‐diversity, we first relativized reads of focal bacteria in each caterpillar. For the abundance‐based null model we multiplied relative abundance by the 16S rRNA copy number quantified with real‐time qPCR (Supporting Information: Methods).

### 2.3 Data Analysis

To quantify the effects of immigration on within‐host gut bacterial community size and α‐diversity, we fit generalized linear mixed effects models with fixed effects for immigration, instar and their interaction. The effect of instar represents changes over time with ontogeny. In all models, immigration was modeled as a continuous variable to test for overall changes in response variables with increasing immigration density (slopes). We included random effects to account for any variation due to individual plants on which caterpillars were reared prior to treatment applications (Plant) and the lineage of individual caterpillars (Lineage). The random effect structure for each response variable, that is, inclusion of random slopes and/or intercepts, was determined by model support (lowest AIC value). We modeled both α‐diversity responses (Richness and 1/Simpson's Dominance) with Gaussian errors and identity link functions, *n* = 78 caterpillars for each model (14 control caterpillars that did not receive microbe immigration treatments are not included). We modeled community size with Poisson distributed error and a log link, which is ideal for count data without overdispersion (confirmed by model fit diagnostics described below). We modeled β‐diversity with beta distributed error and a logit link because it is a continuous response bounded between zero and one. We ensured all models fit the data well using a simulation‐based approach to calculate scaled residuals (Dunn and Smyth 1996; Gelman and Hill 2007), which were normally distributed and uniform across model predictions. We report standardized beta coefficients, confidence intervals, and *p*‐values to put predictors on a common scale and to make main effects interpretable in the presence of interactions (Gelman 2008). We conducted analyses in the statistical software package R version 4.2.2 (R Core Team 2022); we fit general linear mixed models with Restricted Maximum Likelihood and Kenward‐Roger degrees of freedom using lme4 v1.1‐31 (Bates et al. 2015), fit Poisson and Beta mixed effects models with Maximum Likelihood using glmmTMB (Bates et al. 2015), and conducted simulations to assess model fits using DHARMa v0.4.6 (Hartig 2022).

We then used a null‐model randomization to quantify the degree to which the observed β‐diversity among individuals within treatments differed from patterns expected based on stochastic sampling from the treatment microbial pool. Focal bacteria were randomly sampled from the treatment pools with equal probability (because the relative abundance of focal bacteria was equal in each treatment). Bacteria were then assigned to caterpillars within each Immigration × Instar treatment while preserving the observed bacterial richness and community size within each caterpillar (Kraft et al. 2011). Therefore, the number of individuals sampled from the pool into each caterpillar equals the total count of bacteria observed in each caterpillar (determined through qPCR). We repeated this procedure 1000 times to generate the expected null distribution, then calculated standardised effect sizes (β‐deviation) as the difference between the observed β‐diversity and mean expected β‐diversity divided by the standard deviation of the mean expected β‐diversity. Drift is predicted to cause β‐deviations that do not deviate from zero (the null expectation) while more positive or negative β‐deviations indicate greater compositional divergence or convergence than expected from stochastic sampling of the pool (Chase and Myers 2011; Kraft et al. 2011).

## 3 Results

**FIGURE 3:**
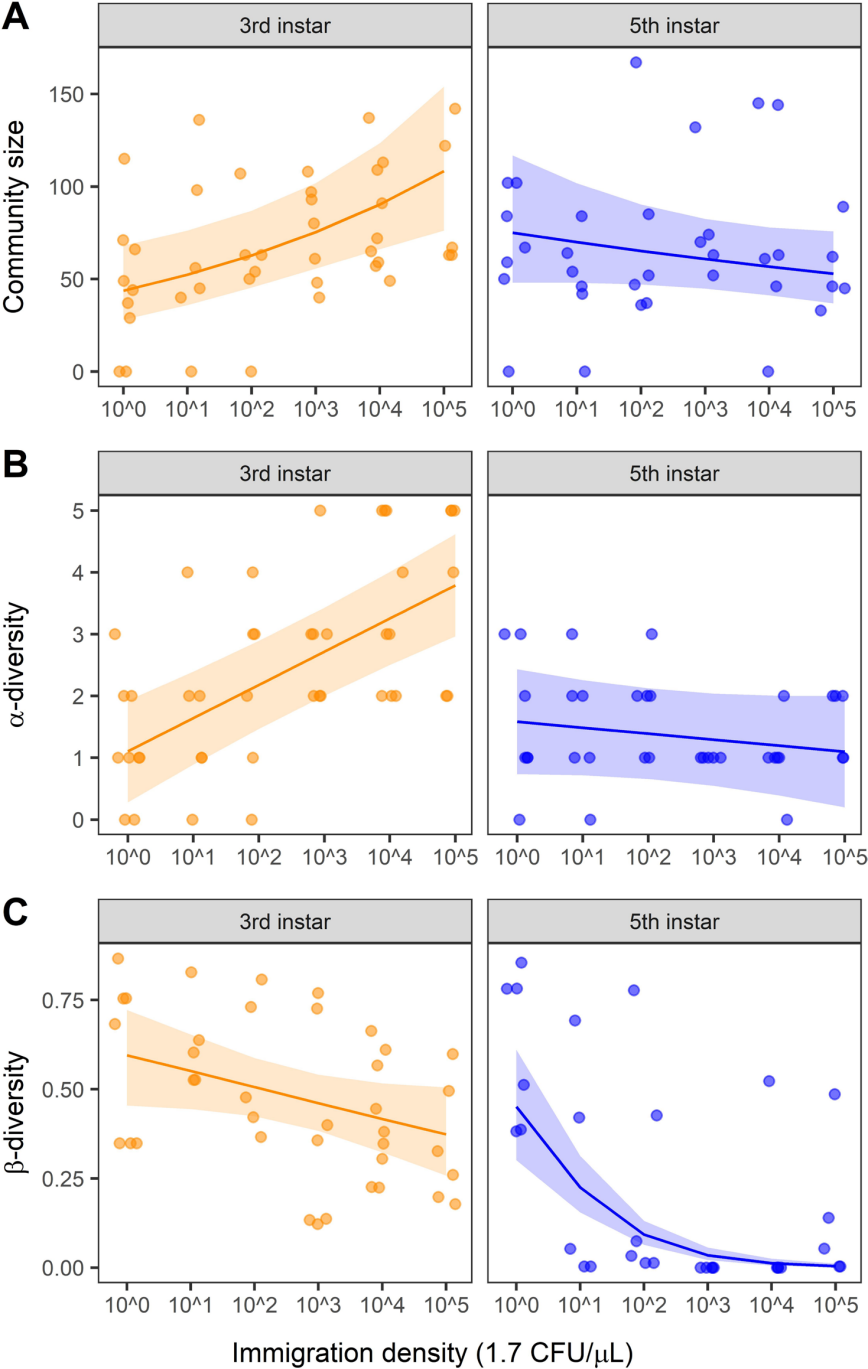
Effects of bacterial immigration on (A) gut community size (abundance of bacteria added from treatments: 16S rRNA copy number per μL; Supporting Information: Methods), (B) α‐diversity (richness of bacteria families from treatments within caterpillars), and (C) β‐diversity (variation in bacteria composition among caterpillars) at two development stages (3rd and 5th instar). Lines are model predictions and their 95% confidence intervals. β‐diversity was calculated as the mean distance‐to‐centroid in principal coordinate space using Bray–Curtis dissimilarity. For visualisation of community size only, one observation was not plotted in panel A (see Figure S1).

Several lines of evidence support the hypothesis that immigration modified the relative importance of niche selection and drift for causing variation in gut microbial composition within and across individuals (Figure 2). First, consistent with the prediction that dispersal increases community size, experimentally increasing bacterial immigration increased the total abundance of bacteria (16S rRNA copy number per μL) from the treatment species pool in caterpillar midguts (Figure 3A, Table S2), thereby meeting the prerequisite condition for higher immigration to mitigate the importance of drift. The positive effect of immigration on bacterial community size at the 3rd instar of development (effect: 0.18, 95% CI [0.08, 0.28]) diminished by the 5th instar (−0.07 [−0.17, 0.03]) (Immigration × Instar std. effect: 0.64 [0.60, 0.69], *p* < 0.001). Mirroring the patterns with community size, immigration increased the number of bacterial taxa (α‐diversity) from the treatment pool that colonised caterpillars (Figure 3B, Figure S2, Table S3), where the positive effect of immigration on α‐diversity at the 3rd instar (0.54 [0.36, 0.71]) diminished by the 5th instar (−0.10 [−0.29, 0.10]) (Immigration × Instar std. effect: −0.81 [−1.14, −0.48], *p* < 0.001). Together, these results suggest that gut microbial assembly is mediated by community size and shifts with ontogeny; from initially being limited by dispersal to being constrained by ecological processes within hosts.

**FIGURE 4:**
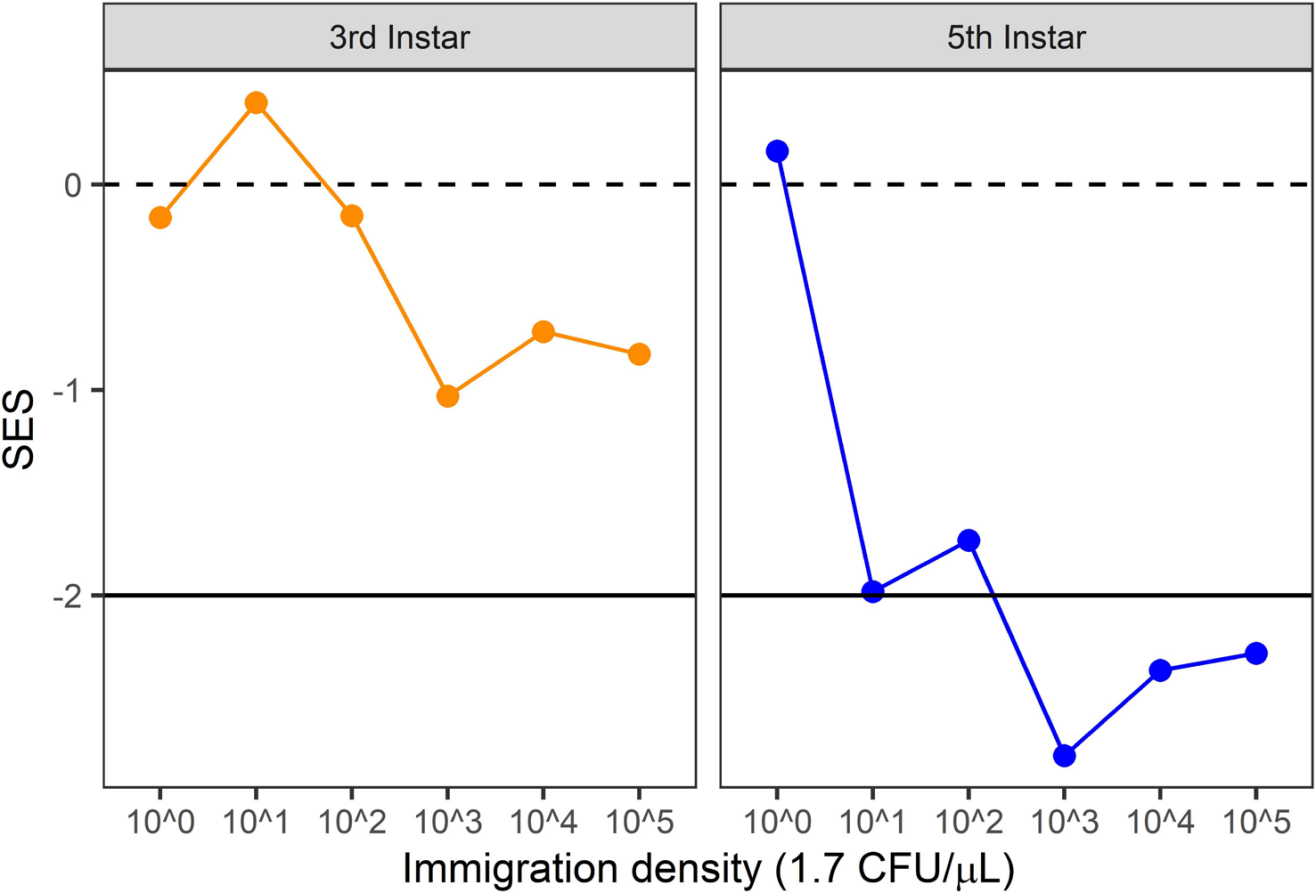
Change in standardised effect size (β‐deviation) within increasing immigration. β‐deviations are the difference between observed β‐diversity and the expected β‐diversity from a null model of stochastic sampling from the treatment pools, divided by the standard deviation of the expected distribution. More negative values indicate gut microbial communities among caterpillars are more similar than expected from stochastic assembly, with SES > 2 indicative of large effects.

Second, increasing immigration consistently reduced bacterial β‐diversity across individual caterpillars; however, the magnitude of convergence in microbial composition increased through ontogeny (Immigration × Instar: std. effect = 0.22 [0.13, 0.40], *p* < 0.001, Figure 3C, Table S4). The negative effect of immigration on β‐diversity at the 3rd instar (−0.18 [−0.36, 0.00]) became stronger by the 5th instar (−1.04 [−1.32, −0.76]). Furthermore, β‐deviations (patterns not due to stochastic sampling from the treatment pools) became increasingly negative by the 5th instar at higher immigration (Figure 4). Both the reductions in observed β‐diversity and null‐model β‐deviations with higher immigration support the hypothesis that dispersal increases the strength of niche selection.

**FIGURE 5:**
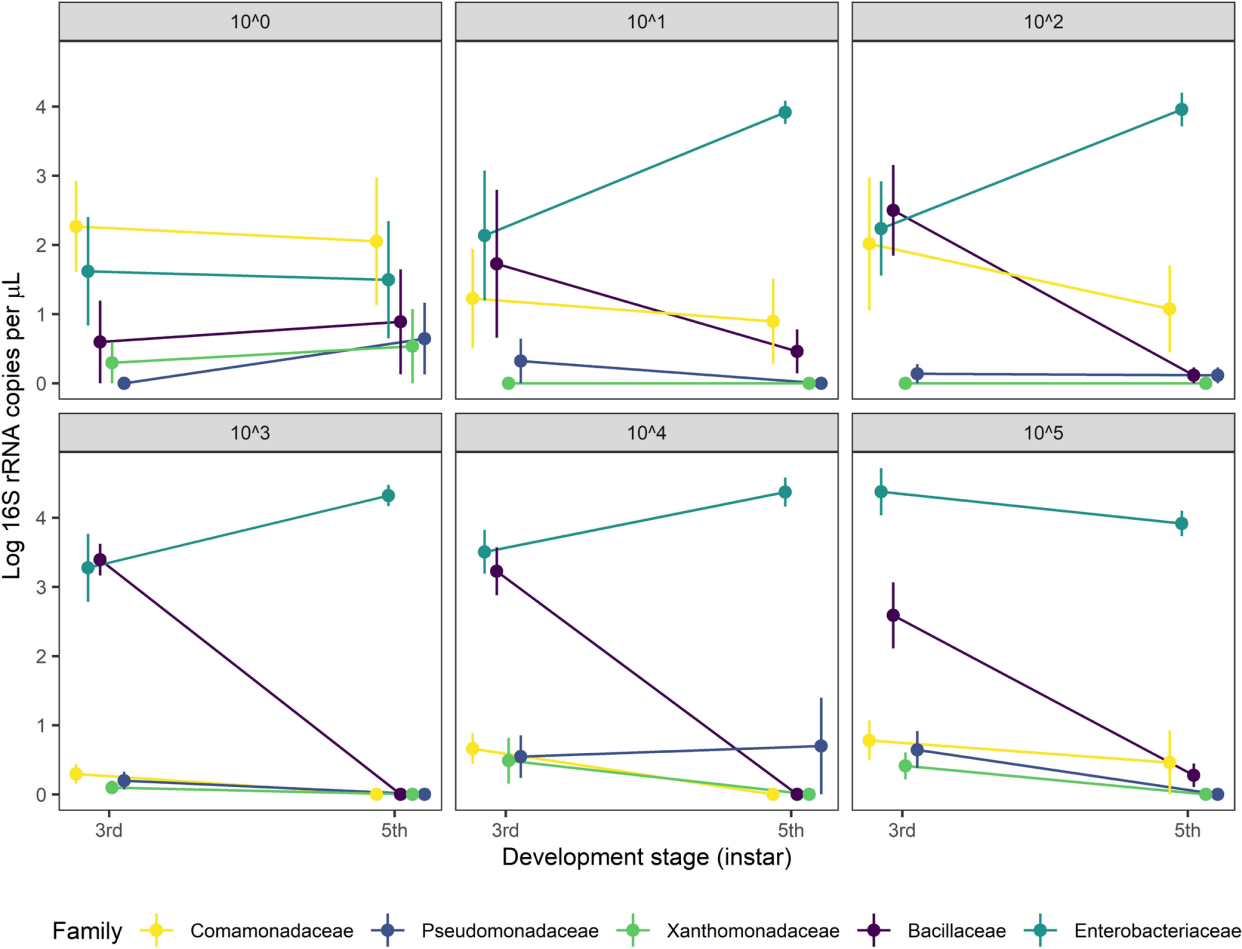
Effects of immigration on change in abundance (log 16S rRNA copy # per μL) of focal bacteria (those added from source pool treatments) through larval development stages (3rd to 5th instar). All 5 taxa were represented in equal relative densities in immigration treatments. Points are mean abundances and their standard deviations calculated across individual caterpillars in each Treatment × Instar combination.

Finally, we found that immigration increased bacterial dominance within caterpillars through ontogeny. Enterobacteriaceae relative abundances increased by 68% in caterpillar guts, from a mean of 48.6% at the 3rd instar to 81.6% by the 5th instar (Figure 2). In almost all individuals, Enterobacteriaceae became increasingly abundant through ontogeny while all other colonising bacteria decreased in abundance (Figure 5). However, Enterobacteriaceae did not consistently colonise individuals in the lowest immigration treatment, thereby increasing opportunities for bacteria that otherwise went locally extinct to maintain stable or positive population growth rates. Importantly, these patterns are not present in treatment controls (Figure 2) demonstrating that changes in abundance reflect population growth or persistence within hosts, not transient dynamics due to repeated acquisition from the environment.

## 4 Discussion

Dispersal is a fundamental process structuring community assembly and biodiversity patterns (Vellend 2010; Leibold et al. 2004; Hubbell 2001), yet experimental evidence from host‐microbe symbiosis is rare (Miller et al. 2018; Nemergut et al. 2013). By constructing experimental bacterial pools of five families—*Enterobacteriaceae* (*Enterobacter* sp.), *Comamonadaceae* (*Delftia* sp.), *Bacillaceae* (*Bacillus* sp.), *Xanthomonadaceae* (*Stenotrophomonas* sp.) *and Pseudomonadaceae* (*Pseudomonas* sp.) and manipulating immigration over six orders of magnitude into monarch caterpillars, we show that higher immigration increased the dominance of certain bacteria, leading to greater convergence in bacterial composition through ontogeny. Conversely, limited immigration made colonisation more stochastic, resulting in greater and more unpredictable variability in bacterial composition across individuals. Thus, immigration from the regional microbial pool increased the importance of niche selection relative to drift in structuring the assembly of caterpillar gut microbial communities.

Several lines of evidence support the hypothesis that bacterial immigration altered the relative importance of niche selection and drift in the assembly of caterpillar gut microbiota. First, higher immigration initially increased community size, α‐diversity within hosts and similarity of bacterial communities across hosts (Figure 3) as more bacterial taxa established in caterpillar guts. The positive relationship between immigration and gut diversity for 3rd instar caterpillars is consistent with studies of macroorganisms showing communities are primarily dispersal limited in early stages of assembly (Catano et al. 2025; Myers and Harms 2009). Second, the increasing importance of niche selection with higher immigration and ontogeny appears to be caused by deterministic outcomes that favor Enterobacteriaceae, such as differential establishment or stronger competition within the caterpillar gut environment. α‐diversity declined with ontogeny as caterpillars transitioned from 3rd to 5th instars and gut microbial communities converged to similar states dominated by the introduced Enterobacteriaceae (Figures 2 and 5). Enterobacteriaceae are commonly found in the caterpillar midgut (Sanaei et al. 2024), yet their commonness resulted from recurring colonization from the external environment (e.g., acquired from the leaf phyllosphere via feeding) or local mechanisms (e.g., competitive interactions within the host gut). While most host‐microbiome research focuses exclusively on local processes operating at the scale of individual hosts (Falony et al. 2016), or in some cases interhost dispersal (Burns et al. 2017), our research demonstrates that variation among hosts is contingent on regional context (species pools and immigration). This result provides experimental evidence consistent with a recent broad‐scale analysis of plant, bee and bee microbiome relationships across biogeographic regions (Argueta‐Guzmán et al. 2025). These results suggest that colonization history and initial community conditions can alter successional trajectories in host‐associated bacterial communities as they often do in macroorganism communities like forests and grasslands (Cadotte 2023).

Moreover, our experiment provides support that higher immigration mediates the strength of niche selection by increasing the number of individuals within the gut of caterpillars, consistent with the effective community size hypothesis (Ron et al. 2018). The effective community size hypothesis predicts that more individuals will increase the strength of deterministic outcomes mediated by species' fitness differences (Orrock and Fletcher Jr. 2005; Hu et al. 2006; Orrock and Watling 2010). For our source pools, we chose bacterial taxa that occur at different relative abundances in caterpillars with the assumption that this reflects differences in their fitness within the caterpillar gut environment. By equalizing their abundances in the source pool, we removed effects due solely to immigration history and regional abundance distributions. Our results show that increasing immigration created initial communities with higher bacterial loads dominated by bacteria from the source pools (Figure 3A, Table S1). Moreover, higher immigration caused greater convergence in bacterial composition than expected by randomly sampling more individuals from the pool (Figure 4).

In contrast, reducing immigration caused smaller initial community sizes (Figure 3A), greater dissimilarity in the microbes that colonised across individuals (Figure 3C), and patterns that are expected by random sampling from the pool (Figure 4). Lower colonisation of Enterobacteriaceae reduced their establishment success (Figure 2) and limited their increase in abundance over time (Figure 5). All other focal bacteria did not establish well or declined in abundance in higher immigration treatments where Enterobacteriaceae were relatively abundant. While low establishment by most taxa, other than Enterobacteriaceae, across immigration treatments suggests an important role of filtering by the gut environment, the capacity for bacteria to maintain higher populations in the absence of Enterobacteriaceae suggests that loss of subordinate taxa under high immigration could also result from competition with Enterobacteriaceae. This result is consistent with metacommunity theory that predicts lower immigration mitigates the spread of the regionally dominant competitor, thus allowing more species to ‘win by forfeit’ (Hurtt and Pacala 1995). However, the interpretation of this specific mechanism should be interpreted cautiously with respect to this mechanism because it is contingent on relatively few caterpillars in the lowest immigration treatment. Also, our analyses cannot reveal the species or strains of bacteria interacting, and ecological interactions like competition will likely occur more strongly at these levels. Future studies, such as reciprocal invasion experiments between hypothesized bacteria competitors (Grainger, Levine, and Gilbert 2019), would be useful to help resolve the specific interactions that promote or erode gut bacterial diversity. Regardless of the lower‐level mechanisms, our study is consistent with a trade‐off predicted by spatial coexistence theory (Shoemaker and Melbourne 2016) and confirmed in plant communities (Catano et al. 2025)–regional constraints on colonisation make assembly more stochastic while also helping to maintain bacterial diversity within the population of caterpillars by limiting bacterial homogenization among hosts.

Surprisingly, despite lower immigration causing bottlenecks in initial community size, we did not find evidence that drift became more important. Following colonisation by microbes from experimental pools, gut microbiota converged over development (Figure 4), inconsistent with models (Orrock and Fletcher Jr. 2005; Orrock and Watling 2010), experiments (Ron et al. 2018; Gilbert and Levine 2017) and correlative studies (Jacobi and Siqueira 2023; Siqueira et al. 2020) that predict lower community size will increase compositional divergence through drift. Though drift is recognised to be an important process in theory (Vellend 2016) and some empirical studies (Gilbert and Levine 2017), its role in more complex host‐associated microbial communities remains rarely quantified (Lankau et al. 2012). While drift appeared to be important at lower immigration densities and early in assembly, niche selection overrode drift even at low community sizes. One potential reason host microbiota appeared less sensitive to drift could be the capacity for bacteria to have faster generation times and achieve larger population sizes than macroorganisms like plants (Nemergut et al. 2013), thereby quickly closing the window where drift is most likely to manifest. Second, the effect of instars is inseparable from the passage of time alone, and caterpillars shedding their gut lining when transitioning to the next instar could impose disturbance that further selects for specific bacteria. Finally, drift is likely more important at the level of species or strains, and family‐level analyses may mask these dynamics. These results underscore the importance of testing theory across divergent systems where generality and idiosyncrasies can be revealed.

Previous research concluded that the microbes in the gut of caterpillars are transient, entirely acquired through their plant diet and incapable of microbial growth due to unfavourable digestive physiology (Hammer et al. 2017). However, our results suggest that some bacteria were able to maintain stable or positive population growth through larval development—persisting through severe disturbances when caterpillars shed their exoskeletal gut lining when moulting (Engel and Moran 2013). While hosts likely exist on a continuum from those with resident and functionally important microbiomes (Bordenstein and Theis 2015; Zilber‐Rosenberg and Rosenberg 2008) to those independent from microbial symbionts, our study suggests regional context (microbial species pools and immigration) can have consequences for the variation in microbial composition within and across hosts. Highly mobile or migratory organisms, like monarchs, will be exposed to different species pools and microbial densities as they span biogeographic regions, ecosystems, and host populations. Therefore, a combination of large‐scale surveys across the monarch range and experimental microbiome assembly across this continuum has the potential to reveal new insights into how ecological context alters host–microbe interactions, which can have broad implications for a general understanding of host ecology and evolution (Moran et al. 2019; McFall‐Ngai et al. 2013). While our lab‐based study provides mechanistic evidence for the importance of even a single immigration event for shaping variation in host‐microbial communities, which can be common in microbiome transplant studies (Greyson‐Gaito et al. 2020; Hartman et al. 2009), future studies would benefit from considering more complex and recurrent dispersal dynamics found in natural systems. Finally, considering broader variation in natural species pools will be important to determine how processes of gut microbial assembly may be contingent on the ecological and evolutionary relatedness of individuals arriving from the pool (Spasojevic et al. 2018; Grainger, Letten, et al. 2019).

## 5 Conclusion

Interest in host‐microbiomes has exploded in recent years with the growing recognition that host microbial communities can mediate broad‐scale patterns of ecological and evolutionary divergence (Moran et al. 2019; McFall‐Ngai et al. 2013) and have important implications for human health and biomedical research (Gilbert et al. 2018; Mazmanian et al. 2008; Bäckhed et al. 2005). However, resolving the surprisingly high variation within and among individual hosts has made it challenging to elucidate the fundamental processes that structure the diversity and distribution of host‐associated microbiota. We show that a tighter integration between multiscale ecological theory and experiments has the potential to greatly increase our understanding of host‐microbe symbiosis. More generally, our study suggests that the consequences of dispersal on the tension between ecological selection and drift (Catano et al. 2025; Germain et al. 2017) are a general phenomenon governing the assembly and distribution of biodiversity across domains of life.

## Author Contributions

Christopher P. Catano and Jacobus C. de Roode designed research. Christopher P. Catano, Joselyne Chavez, Lydia Fuller‐Hall and James G. DuBose performed research. James G. DuBose processed DNA sequences. Christopher P. Catano analyzed data. Christopher P. Catano wrote the first draft. All authors contributed to revisions.

## Supporting information


**Data S1:** ele70252‐sup‐0001‐SupplementalMethods.docx.

**Data S2:** ele70252‐sup‐0002‐DataS2.docx.docx.

## Data Availability

All data and code necessary to reproduce the results and figures in this paper are freely available on GitHub and Zenodo.org: https://doi.org/10.5281/zenodo.17307648. DNA sequences have been deposited into the NCBI SRA database and can be accessed using the BioProject ID PRJNA1106479.
