# Supplementary material for: Experimental Immigration Mediates Ecological Selection and Drift in Monarch Microbiome Assembly
Source: Ecol Lett. 2025 Nov 9;28(11):e70252. doi: 10.1111/ele.70252 (PMC12597025; doi:10.1111/ele.70252)
Supplement: Supplementary file 1 — Data S1: ele70252‐sup‐0001‐SupplementalMethods.docx. [file ELE-28-0-s001.docx]

**Supplemental Methods**

*DNA sequencing & processing*: We determined caterpillar gut bacterial community composition and changes over time by sequencing bacterial DNA from caterpillar midguts. We surface-sterilized caterpillars in 80% ethanol then rinsed with Ultrapure™ distilled water (Invitrogen). Next, we then removed and transferred the midguts into 200-μl of sterile 1×PBS and stored them at -20°C. Prior to extraction, we homogenized the tissue in the lysis buffer using sterile pestles to increase yield. We extracted DNA from the midguts using the Qiagen DNeasy PowerSoil Pro kit and sterile techniques. Due to the extensive amount of tissue, the 5^th^ instar midgut DNA extractions were performed in 2-4 reactions, all completed within the same extraction batch. We assessed quantity and quality of the DNA extracts using a NanoDrop™ One Spectrophotometer. We sent extracted DNA to the University of Georgia Genomics Core for 300bp paired-end sequencing on an Illumina MiSeq. Library preparation was performed by the sequencing facility using the 341f and 785r primers, which target the V3-V4 region of the 16S rRNA gene (Klindworth *et al.* 2013). Raw sequences can be accessed from the NCBI SRA database and using the BioProject ID PRJNA1106479.

To process bacterial DNA sequences we first removed adapter, primer, low quality bases, and chimeric sequences using the Divisive Amplicon Denoising Algorithm (DADA2) (Callahan *et al.* 2016). The ends of each read were trimmed at the position where the median phred score dropped below 20. Reads with at least 12 base-pairs of identical overlap were then merged and reads that were not able to be merged were discarded. To improve the resolution of taxonomic assignment, we used the REference Sequence annotation and CuRatIon Pipeline (RESCRIPt) (Robeson *et al.* 2021) to curate an amplicon-specific form (V3-V4 region) of the SILVA SSU NR99 database (v.138.1). We then used the q2-feature-classifier plugin (Bokulich *et al.* 2018) to train a Naïve Bayes classifier on the curated database, as well as assign taxonomy to each amplicon sequence variant (ASV). ASVs that were classified as either mitochondria or chloroplast were discarded (Fig. S3). We then used the q2-quality-control plugin (Bolyen *et al.* 2019) to align each sequence to the previously described curated database. Sequences that did not have an alignment of at least 80% identity across 80% of any reference sequence were discarded, totaling approximately 848 ASVs that constituted approximately 6.6% of the total reads across samples. After sequence processing, we retained an average of 24,459 (std. dev. = 19,059) sequences per sample. Sequencing error rates limited our capacities to accurately quantify abundance of ASVs representing focal taxa when rare, which is especially likely in the lowest immigration treatments). Therefore, we use a more conservative approach by aggregating bacteria to the family level (ASVs representing isolates of focal bacteria represented 77% of the reads within their respective families across treatments. We used the collapse function in the q2-taxa plugin (Bolyen *et al.* 2019) to generate family counts. We performed all sequence processing using QIIME2 (v. 2023.2).

*Real-time qPCR:* We quantified gut community size as the 16S rRNA copy number per μL of extracted DNA with real-time qPCR. We used primers and instrument parameters targeting the 16S rRNA V4 region (Carini *et al.* 2017). We used an IDT g-block™, a synthetic gene fragment of the V4 region of the *E. Coli* substrain DH10β ([Accession CP000948](https://rnacentral.org/rna/URS00000ABFE9/316385)), which has seven 16S rRNA operons per genome (Durfee *et al.* 2008), as the reference standard. For confirmation the g-block concentration was measured in triplicate via NanoDrop: 16.104 ± 0.2 ng/μl, which equivalates to 5.168 x 10^10^ copies per μl. 10^-6^ to 10^-9^ dilutions of the g-block were used to generate the reference standard curve. Primers were tested for efficiency and specificity (slope = -3.45, efficiency = 94.98%, R^2^ = 0.99). An Applied Biosystems StepOnePlus Real-time PCR system was utilized for the quantification. The components of reaction consisted of 5μl Bio-Rad’s SsoAdvanced™ Universal SYBR® Green Supermix, 1μl 515f primer (3μM), 1μl 806r primer (3μM), 1μl Invitrogen Ultrapure Nuclease-free distilled water and 2μl template. Plates were spun down for 3 mins at 1500 rpm. The samples were run in triplicate with an additional Melt Curve analysis for each run. The templates were normalized to 10 ng/μl (concentration was determined based on standard curves).

**Primers:**

16S rRNA V3V4 (Klindworth *et al.* 2013)

341f: 5’-CCTACGGGNGGCWGCAG-3’

785r: 5’-GACTACHVGGGTATCTAATCC-3’

16S rRNA V4 region primers (Carini *et al.* 2017)

515f: 5′-GTGCCAGCMGCCGCGGTAA-3′

806r: 5′-GGACTACHVGGGTWTCTAAT-3′

16S rRNA gene (Frank *et al.* 2008; Monciardini *et al.* 2002)

27f: 5’-AGAGTTTGATCMTGGCTCAG-3’

1492r: 5’-GGTTACCTTGTTACGACTT-3’

**Reference Standard:** IDT g-block derived from Escherichia coli str. K-12 substr. DH10β bacterial 16S SSU rRNA ([Accession CP000948](https://rnacentral.org/rna/URS00000ABFE9/316385)) – JC in-house design

>SYN V4 DH108B TAACTCCGTGCCAGCAGCCGCGGTAATACGGAGGGTGCAAGCGTTAATCGGAATTACTGGGCGTAAAGCGCACGCAGGCGGTTTGTTAAGTCAGATGTGAAATCCCCGGGCTCAACCTGGGAACTGCATCTGATACTGGCAAGCTTGAGTCTCGTAGAGGGGGGTAGAATTCCAGGTGTAGCGGTGAAATGCGTAGAGATCTGGAGGAATACCGGTGGCGAAGGCGGCCCCCTGGACGAAGACTGACGCTCAGGTGCGAAAGCGTGGGGAGCAAACAGGATTAGATACCCTGGTAGTCCACGCC

**References:**

Bokulich, N.A., Kaehler, B.D., Rideout, J.R., Dillon, M., Bolyen, E., Knight, R., *et al.* (2018). Optimizing taxonomic classification of marker-gene amplicon sequences with QIIME 2’s q2-feature-classifier plugin. *Microbiome*, 6, 90.

Bolyen, E., Rideout, J.R., Dillon, M.R., Bokulich, N.A., Abnet, C.C., Al-Ghalith, G.A., *et al.* (2019). Reproducible, interactive, scalable and extensible microbiome data science using QIIME 2. *Nat Biotechnol*, 37, 852–857.

Callahan, B.J., McMurdie, P.J., Rosen, M.J., Han, A.W., Johnson, A.J.A. & Holmes, S.P. (2016). DADA2: High-resolution sample inference from Illumina amplicon data. *Nat Methods*, 13, 581–583.

Carini, P., Marsden, P.J., Leff, J.W., Morgan, E.E., Strickland, M.S. & Fierer, N. (2017). Relic DNA is abundant in soil and obscures estimates of soil microbial diversity. *Nat Microbiol*, 2, 16242.

Durfee, T., Nelson, R., Baldwin, S., Plunkett, G., Burland, V., Mau, B., *et al.* (2008). The Complete Genome Sequence of Escherichia coli DH10B: Insights into the Biology of a Laboratory Workhorse. *Journal of Bacteriology*, 190, 2597–2606.

Frank, J.A., Reich, C.I., Sharma, S., Weisbaum, J.S., Wilson, B.A. & Olsen, G.J. (2008). Critical Evaluation of Two Primers Commonly Used for Amplification of Bacterial 16S rRNA Genes. *Appl Environ Microbiol*, 74, 2461–2470.

Klindworth, A., Pruesse, E., Schweer, T., Peplies, J., Quast, C., Horn, M., *et al.* (2013). Evaluation of general 16S ribosomal RNA gene PCR primers for classical and next-generation sequencing-based diversity studies. *Nucleic Acids Research*, 41, e1–e1.

Monciardini, P., Sosio, M., Cavaletti, L., Chiocchini, C. & Donadio, S. (2002). New PCR primers for the selective amplification of 16S rDNA from different groups of actinomycetes1. *FEMS Microbiology Ecology*, 42, 419–429.

Robeson, M.S., O’Rourke, D.R., Kaehler, B.D., Ziemski, M., Dillon, M.R., Foster, J.T., *et al.* (2021). RESCRIPt: Reproducible sequence taxonomy reference database management. *PLoS Comput Biol*, 17, e1009581.
