# Supplementary material for: Experimental Immigration Mediates Ecological Selection and Drift in Monarch Microbiome Assembly
Source: Ecol Lett. 2025 Nov 9;28(11):e70252. doi: 10.1111/ele.70252 (PMC12597025; doi:10.1111/ele.70252)
Supplement: Supplementary file 2 — Data S2: ele70252‐sup‐0002‐DataS2.docx.docx. [file ELE-28-0-s002.docx]

**Supplemental Results**

**Table S1.** Total proportion of taxa in the gut microbial communities represented by the focal bacteria added with each immigration treatment. The mean and standard deviation are calculated across all individual caterpillars within each Instar x Treatment group.

| **Instar** | **Treatment** | **Mean** | **SD** |
| --- | --- | --- | --- |
| 3rd | Control | 0.11 | 0.28 |
|  | 1.7x10^0 | 0.14 | 0.23 |
|  | 1.7x10^1 | 0.49 | 0.48 |
|  | 1.7x10^2 | 0.45 | 0.45 |
|  | 1.7x10^3 | 0.58 | 0.44 |
|  | 1.7x10^4 | 0.77 | 0.31 |
|  | 1.7x10^5 | 0.95 | 0.05 |
|  |  |  |  |
| 5th | Control | 0.13 | 0.23 |
|  | 1.7x10^0 | 0.21 | 0.23 |
|  | 1.7x10^1 | 0.44 | 0.35 |
|  | 1.7x10^2 | 0.45 | 0.42 |
|  | 1.7x10^3 | 0.6 | 0.38 |
|  | 1.7x10^4 | 0.49 | 0.41 |
|  | 1.7x10^5 | 0.54 | 0.39 |

**Table S2.** Outcomes of bacterial community size (total abundance: 16S rRNA copy number per μL extracted DNA) that experimentally colonized monarch caterpillar guts. Treatment effects were quantified with a generalized linear mixed model with Poisson distribution and log-link, with random slopes (Immigration) and intercepts for caterpillar lineage and the individual plant on which caterpillars were reared. All results reported below are of standardized beta-coefficients, CIs, and *p*-values.

|  | **Gut bacterial community size** | | | |
| --- | --- | --- | --- | --- |
| *Predictors* | *Coefficient* | *std. Error* | *95% CI* | *p* |
| (Intercept) | 67.52 | 10.75 | 49.42 – 92.25 | **<0.001** |
| Immigration density | 1.37 | 0.12 | 1.15 – 1.64 | **<0.001** |
| Instar [5^th^ instar] | 0.94 | 0.03 | 0.89 – 1.00 | **0.035** |
| Immigration × Instar | 0.64 | 0.02 | 0.60 – 0.69 | **<0.001** |
| **Random Effects** | | | | |
| σ^2^ | 0.01 | | | |
| τ_00_ _Plant_ | 0.26 | | | |
| τ_00_ _Lineage_ | 0.05 | | | |
| τ_11_ _Immigration \| Plant_ | 0.01 | | | |
| τ_11_ _Immigration \| Lineage_ | 0.00 | | | |
| ρ_01_ _Plant_ | -0.89 | | | |
| ρ_01_ _Lineage_ | -0.43 | | | |
| ICC | 0.95 | | | |
| N _Plant_ | 7 | | | |
| N _Lineage_ | 4 | | | |
| Observations | 78 | | | |
| Marginal R^2^ / Conditional R^2^ | 0.25 / 0.96 | | | |

**Table S3.** Outcomes of bacterial α-diversity (Richness; Hill Diversity 0) that experimentally colonized monarch caterpillar guts. Treatment effects were quantified with a linear mixed model fit by Restricted Maximum Likelihood with random intercepts for caterpillar lineage and the individual plant on which caterpillars were reared. All results reported below are of standardized beta-coefficients, CIs, and *p*-values (data centered and scaled prior to fitting model).

|  | **Gut bacterial α-diversity (Richness)** | | | | | | | | |
| --- | --- | --- | --- | --- | --- | --- | --- | --- | --- |
| *Predictors* | *Coefficient* | *std. Error* | *95% CI* | *p* | |  |  |  |  |
| (Intercept) | 0.27 | 0.26 | -0.25 – 0.79 | 0.304 | |  |  |  |  |
| Immigration density | 0.69 | 0.11 | 0.47 – 0.90 | **<0.001** | |  |  |  |  |
| Instar [5^th^] | -0.76 | 0.16 | -1.08 – -0.44 | **<0.001** | |  |  |  |  |
| Immigration × Instar | -0.81 | 0.16 | -1.14 – -0.48 | **<0.001** | |  |  |  |  |
| **Random Effects** | | | | | | | | | |
| σ^2^ | 0.92 | | | | | | | | |
| τ_00_ _Plant_ | 0.07 | | | | | | | | |
| τ_00_ _Lineage_ | 0.35 | | | | | | | | |
| ICC | 0.31 | | | | | | | | |
| N _Plant_ | 7 | | | | | | | | |
| N _Lineage_ | 4 | | | | | | | | |
| Observations | 78 | | | | | | | | |
| Marginal R^2^ / Conditional R^2^ | 0.37 / 0.57 |  |  |  |  | |  |  |  |

**Table S4.** Outcomes of β-diversity (variation in microbial composition among individuals) that experimentally colonized monarch caterpillar guts. Treatment effects were quantified with a generalized linear mixed model with beta distribution and logit-link, with random intercepts for caterpillar lineage and the individual plant on which caterpillars were reared. All results reported below are of standardized beta-coefficients, CIs, and *p*-values.

|  | **Gut bacterial β-diversity** | | | | | | | | |
| --- | --- | --- | --- | --- | --- | --- | --- | --- | --- |
| *Predictors* | *Coefficient* | *std. Error* | *95% CI* | *p* | |  |  |  |  |
| (Intercept) | 0.93 | 0.15 | 0.68 – 1.27 | 0.663 | |  |  |  |  |
| Immigration density | 0.73 | 0.12 | 0.53 – 1.00 | 0.053 | |  |  |  |  |
| Instar [5^th^] | 0.06 | 0.02 | 0.04 – 0.11 | **<0.001** | |  |  |  |  |
| Immigration × Instar | 0.22 | 0.06 | 0.13 – 0.40 | **<0.001** | |  |  |  |  |
| **Random Effects** | | | | | | | | | |
| σ^2^ | 0.53 | | | | | | | | |
| τ_00_ _Plant_ | 0.00 | | | | | | | | |
| τ_00_ _Lineage_ | 0.00 | | | | | | | | |
| N _Plant_ | 7 | | | | | | | | |
| N _Lineage_ | 4 | | | | | | | | |
| Observations | 71 | | | | | | | | |
| Marginal R^2^ / Conditional R^2^ | 0.86 / NA |  |  |  |  | |  |  |  |

**Table S5**. Outcomes of bacterial α-diversity (Hill Diversity 2) that experimentally colonized monarch caterpillar guts. Treatment effects were quantified with a linear mixed model fit by Restricted Maximum Likelihood with random intercepts for caterpillar lineage and the individual plant on which caterpillars were reared. Immigration was centered and scaled prior to model fitting and Estimates, std. Error, 95% CIs, and p-values are thus standardized.

|  | **Gut bacterial α-diversity (Hill Diversity 2)** | | | | |
| --- | --- | --- | --- | --- | --- |
| *Predictors* | *Estimates* | *std. Error* | *CI* | *p* |  |
| (Intercept) | 1.27 | 0.19 | 0.89 – 1.64 | **<0.001** |  |
| Immigration density | 0.31 | 0.07 | 0.17 – 0.44 | **<0.001** |  |
| Instar [5^th^] | -0.26 | 0.10 | -0.46 – -0.07 | **0.010** |  |
| Immigration × Instar | -0.34 | 0.10 | -0.54 – -0.14 | **0.001** |  |
| **Random Effects** | | | | | |
| σ^2^ | 0.19 | | | | |
| τ_00_ _Plant_ | 0.00 | | | | |
| τ_00_ _Lineage_ | 0.12 | | | | |
| ICC | 0.39 | | | | |
| N _Plant_ | 7 | | | | |
| N _Lineage_ | 4 | | | | |
| Observations | 78 | | | | |
| Marginal R^2^ / Conditional R^2^ | 0.185 / 0.506 | | | | |


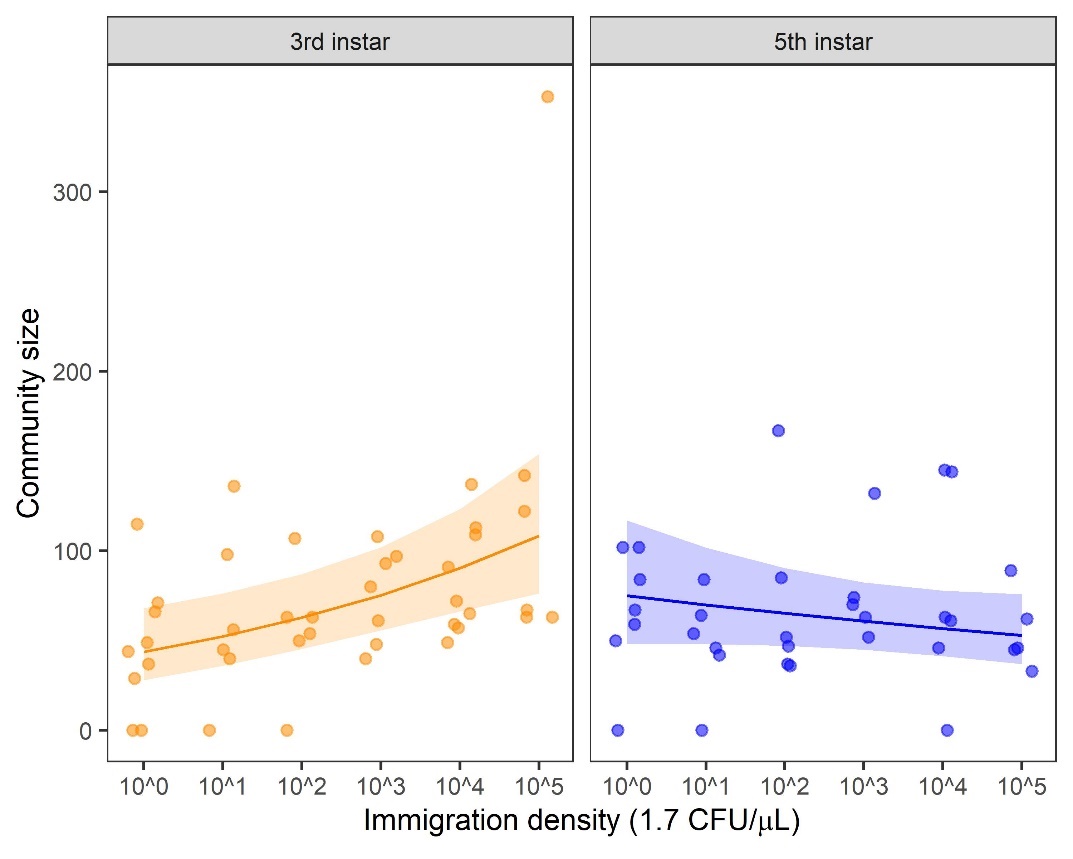


**Figure S1.** Effects of bacterial immigration on gut bacterial community size (bacterial abundance: 16S rRNA copy number per μL: Supplemental Methods) at two development stages (3^rd^ and 5^th^ instar). Lines are model predictions and their 95% confidence intervals. The outlying point in the 10^5^ Immigration treatment was removed from the plot in Fig 3 to aid visualization of model predictions; however, the significance of the model coefficients does not change (*p*-values remain < 0.05) regardless of inclusion or exclusion of this observation.


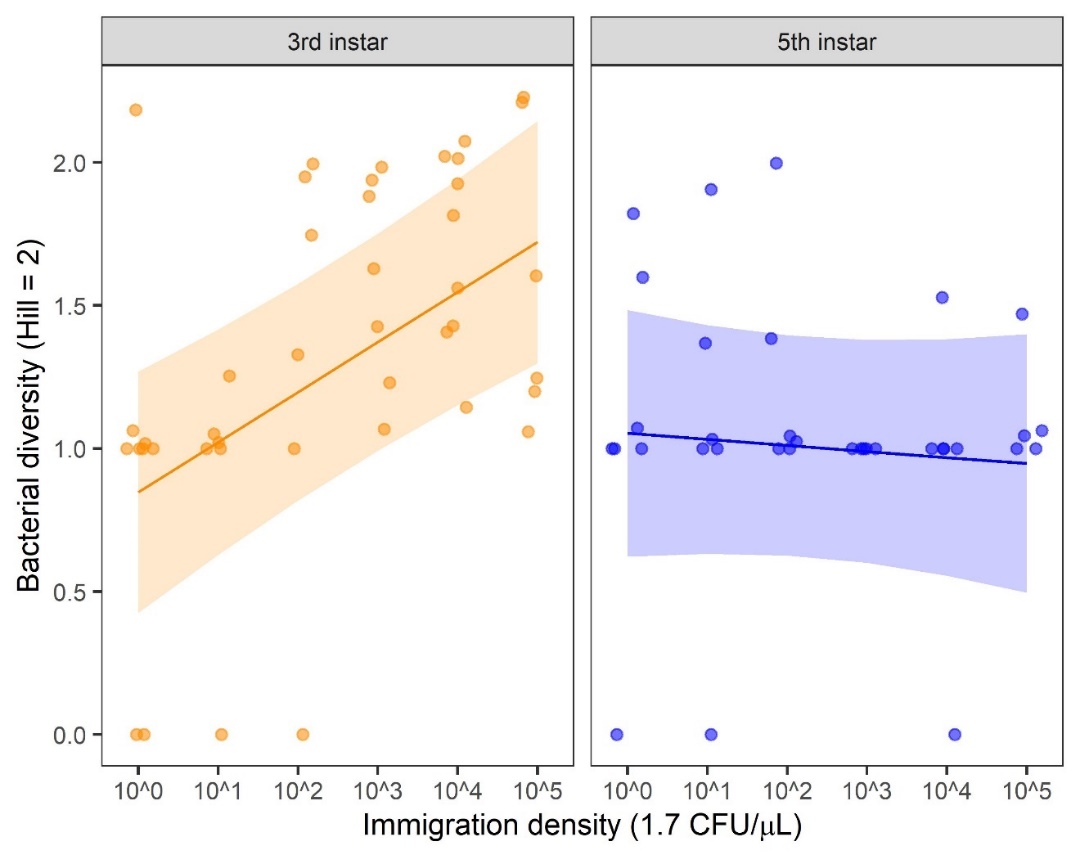


**Figure S2**. Outcomes of bacterial α-diversity (Hill Diversity 2) that experimentally colonized monarch caterpillar guts. Treatment effects were quantified with a linear mixed model fit by Restricted Maximum Likelihood with random intercepts for caterpillar lineage and the individual plant on which caterpillars were reared. Immigration was centered and scaled prior to model fitting and Estimates, std. Error, 95% CIs, and p-values are thus standardized (*n* = 78; Table S2).


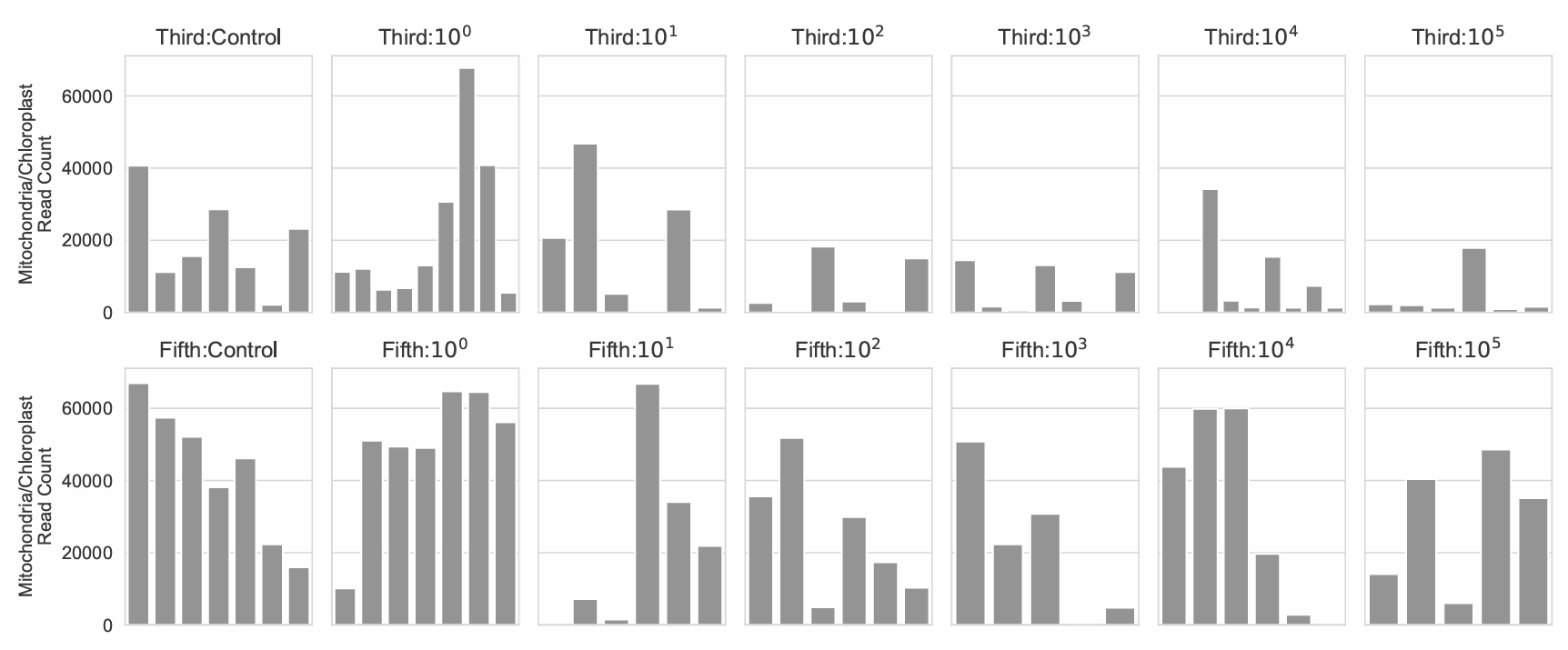


**Figure S3**. Number of Mitochondria and chloroplast reads that were discarded for each caterpillar in each immigration treatment. Third and Fifth correspond to the development stage of the individual (instar). 10^x^ corresponds to the multiplier for the immigration density (1.7 CFU/μL).


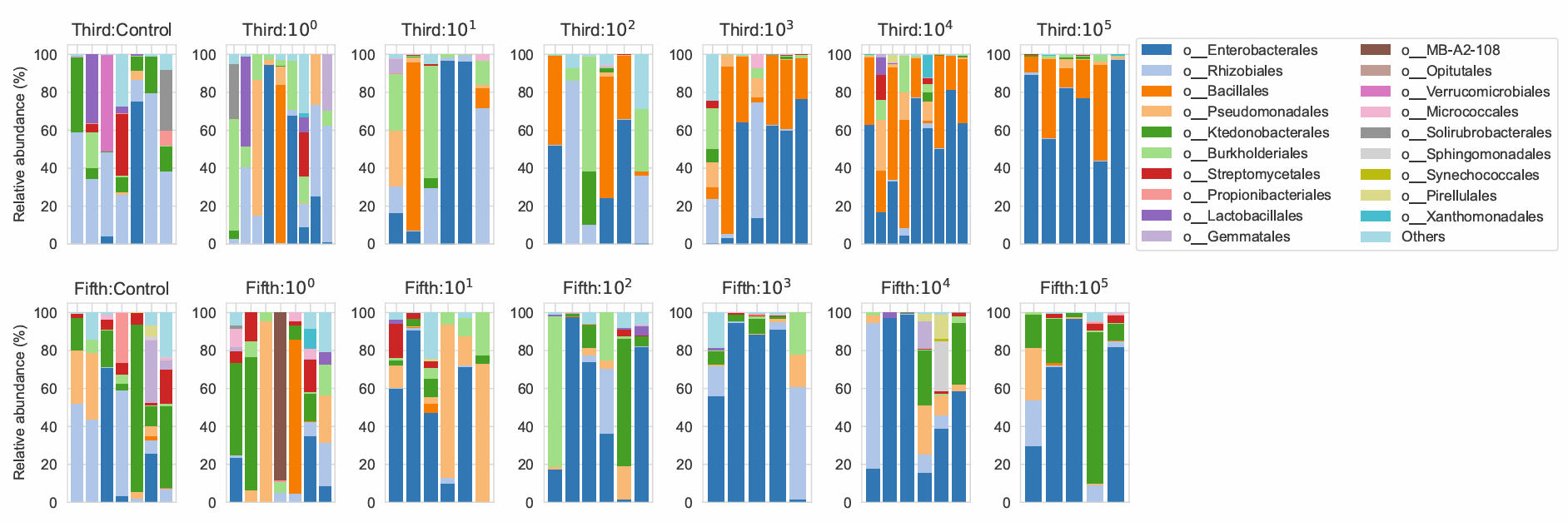


**Figure S4**. Taxa bar plot depicting the relative abundance of each order of bacteria in each individual caterpillar, including those that we added through immigration treatments and those that colonized passively from the environment. Third and Fifth correspond to the development stage of the individual (instar). 10^x^ corresponds to the multiplier for the immigration density (1.7 CFU/μL).
